# Supplementary material for: Possible Link between Higher Transmissibility of Alpha, Kappa and Delta Variants of SARS-CoV-2 and Increased Structural Stability of Its Spike Protein and hACE2 Affinity
Source: Int J Mol Sci. 2021 Aug 24;22(17):9131. doi: 10.3390/ijms22179131 (PMC8431609; doi:10.3390/ijms22179131)
Supplement: Supplementary file 1 [file ijms-22-09131-s001.zip › ijms-1285463-supplementary.pdf]

Possible link between higher transmissibility of Alpha, Kappa and Delta variants of SARS-CoV-2 and increased structural stability of its spike protein and hACE2 affinity

Vipul Kumar[1], Jasdeep Singh[1], Seyed E. Hasnain[1,2,*] and Durai Sundar[1,*]

[1]Department of Biochemical Engineering and Biotechnology, Indian Institute of Technology (IIT) Delhi, New Delhi 110016, India; vipul.kumar@debeb.iitd.ac.in (V.K)

[2]JH-Institute of Molecular Medicine, Jamia Hamdard, New Delhi 110062, India; jasdeep002@gmail.com (J.S)

[3]Department of Life Science, School of Basic Sciences and Research, Sharda University, Greater Noida 201301, Uttar Pradesh, India

*Correspondence: seh@dbeb.iitd.ac.in (S.E.H), sundar@dbeb.iitd.ac.in (D.S)

Supplementary Figures

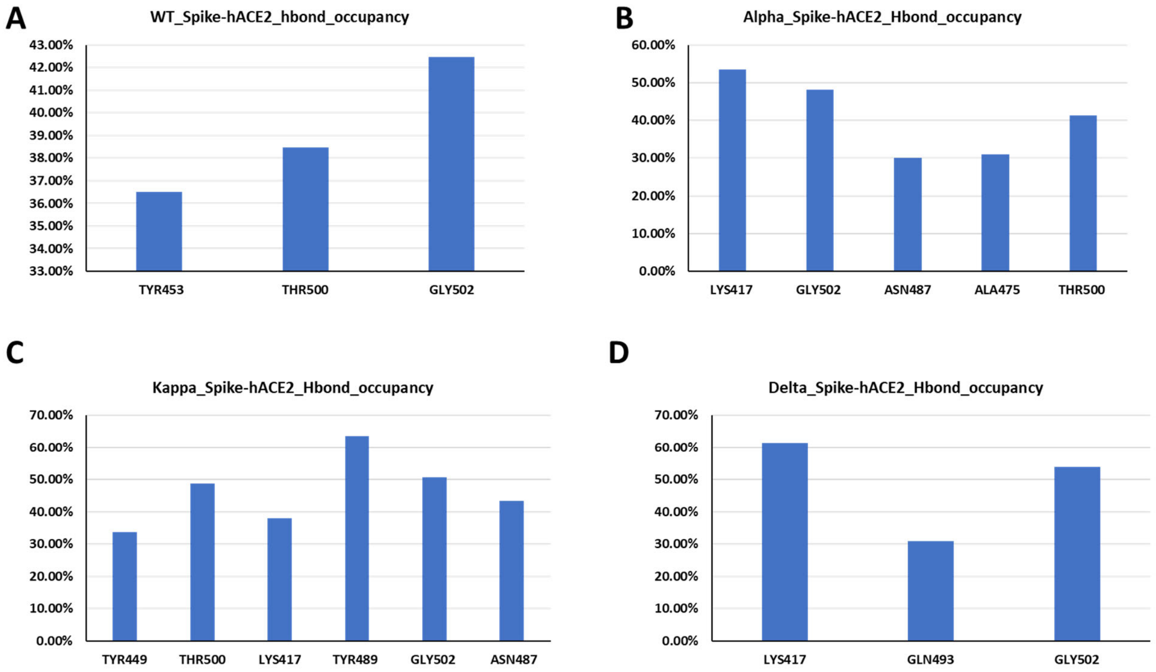

Figure S1. (A) The hydrogen bond occupancy in WT Spike-hACE2 complex, (B) Alpha variant (C) Kappa and (D) Delta variant throughout the 200ns of MD simulations.
